# What Makes Organoids Good Models of Human Neurogenesis?

**DOI:** 10.3389/fnins.2022.872794

**Published:** 2022-04-14

**Authors:** Qian Yang, Yan Hong, Ting Zhao, Hongjun Song, Guo-li Ming

**Affiliations:** ^1^Department of Neuroscience and Mahoney Institute for Neurosciences, Perelman School of Medicine, University of Pennsylvania, Philadelphia, PA, United States; ^2^Department of Cell and Developmental Biology, Perelman School of Medicine, University of Pennsylvania, Philadelphia, PA, United States; ^3^Institute for Regenerative Medicine, University of Pennsylvania, Philadelphia, PA, United States; ^4^The Epigenetics Institute, Perelman School of Medicine, University of Pennsylvania, Philadelphia, PA, United States; ^5^Department of Psychiatry, Perelman School of Medicine, University of Pennsylvania, Philadelphia, PA, United States

**Keywords:** brain organoids, neurogenesis, neural development, stem cell, induced pluripotent stem cells

## Abstract

Human neurogenesis occurs mainly in embryonic, fetal, and neonatal stages and generates tremendously diverse neural cell types that constitute the human nervous system. Studies on human neurogenesis have been limited due to a lack of access to human embryonic and fetal tissues. Brain organoids derived from human pluripotent stem cells not only recapitulate major developmental processes during neurogenesis, but also exhibit human-specific features, thus providing an unprecedented opportunity to study human neurodevelopment. First, three-dimensional brain organoids resemble early human neurogenesis with diverse stem cell pools, including the presence of primate-enriched outer radial glia cells. Second, brain organoids recapitulate human neurogenesis at the cellular level, generating diverse neuronal cell types and forming stratified cortical layers. Third, brain organoids also capture gliogenesis with the presence of human-specific astrocytes. Fourth, combined with genome-editing technologies, brain organoids are promising models for investigating functions of human-specific genes at different stages of human neurogenesis. Finally, human organoids derived from patient iPSCs can recapitulate specific disease phenotypes, providing unique models for studying developmental brain disorders of genetic and environmental causes, and for mechanistic studies and drug screening. The aim of this review is to illustrate why brain organoids are good models to study various steps of human neurogenesis, with a focus on corticogenesis. We also discuss limitations of current brain organoid models and future improvements.

## Introduction

The human brain is one of the most complex and challenging organs to study. Higher intellectual and cognitive functions require all neural cell types with appropriate quantities and properties to form intricate networks for information processing. Neurogenesis, a process starting with the proliferation of neural stem cells and progenitor cells, followed by differentiation into neurons, is the foundation of brain development and function. Due to ethical reasons, accessibility to embryonic and fetal tissue is limited and it is difficult to apply many experimental approaches to primary human brain tissue. Thus, many key questions remain to be answered in human neurogenesis.

Recent advances in three dimensional (3D) organoid culture provide unique tools to overcome hurdles in studying human neurogenesis (Eiraku et al., 2008; Kadoshima et al., 2013; Lancaster et al., 2013; Qian et al., 2016). Brain organoids are self-organized cell aggregates derived from stem cells or induced pluripotent stem cells (iPSCs) that mimic fetal brain development. They have the potential to generate different brain cell types and structures relying on both intrinsic signals and external patterning cues (Lancaster and Knoblich, 2014; Qian et al., 2016; Monzel et al., 2017; Renner et al., 2017; Xiang et al., 2017). Using factors related to developmental patterning signals, organoids can be directed to model brain regions including the cortex, hippocampus, ganglionic eminences, thalamus, hypothalamus and cerebellum (Lancaster et al., 2013; Muguruma et al., 2015; Sakaguchi et al., 2015; Qian et al., 2016; Bagley et al., 2017; Birey et al., 2017; Monzel et al., 2017; Xiang et al., 2019; Huang et al., 2021), and even brain subregions, such as the arcuate nucleus of the hypothalamus (Huang et al., 2021). This model provides the opportunity to understand not only evolutionarily conserved, but also late evolved human-specific features of brain development as well as neurodevelopmental disorders. We focus on cortical organoids as an example as they are most studied.

## Classic Models Studying Neurogenesis

Most of our understanding of neurogenesis comes from studies using *in vivo* models of flies, fish and rodents, which have contributed invaluable insights into neurogenic processes. Among these, the mouse is the most widely used animal model given its many conserved features with human neurogenesis (Table 1). The availability of diverse genetic tools in mice has been a great advantage, which allows scientists to manipulate evolutionally conserved genes or introduce human-specific genes essential for neurogenesis (Mira and Morante, 2020; Navabpour et al., 2020). Decades of studies in rodent models have led to the identification of mechanisms underlying many key features of neurogenesis, such as patterning cues, stem cell proliferation, neural cell production and migration, and circuitry formation. However, mouse models lack human-specific features critical for human neurogenesis. During evolution, the cerebral cortex of the human brain, which contributes to higher cognitive functions and distinguished linguistic abilities, has greatly expanded (Taverna et al., 2014; Hartenstein and Stollewerk, 2015). Human-specific genes and cell types are major contributors to this unique neurodevelopmental process (Bakken et al., 2016; O’Neill et al., 2018; Hodge et al., 2019; Prodromidou and Matsas, 2019; Benito-Kwiecinski et al., 2021). Moreover, mice are lissencephalic and lack the folded neocortical surface as in primates and humans (Rakic, 2009; Fietz et al., 2010; Hansen et al., 2010; Lui et al., 2011; Betizeau et al., 2013; Hartenstein and Stollewerk, 2015; Liu and Silver, 2021). Furthermore, due to differences in species-specific genes and gene regulation, mice are not always a reliable model for human disorders and translational studies (Denayer et al., 2014).

**TABLE 1:** Comparison of organoid, mouse and 2D cell culture models.

	Organoid	Mouse	2D cell culture
Cell types	Heterogeneous	Heterogeneous, rodent specific	Homogenous/heterogeneous
Tissue architecture	Conserved	Conserved, rodent specific	Lost
Cell-cell interaction	Conserved	Conserved	Lost
Temporal order	Conserved	Conserved	Conserved
Human specific cell type	Conserved	Lost	Somewhat conserved
Human specific gene	Conserved	Can be introduced by genetic tools, but in rodent genetic context	Conserved
Disease modeling	Good	Moderate	Moderate
Reproducibility	Low	High	Moderate
Technical consideration	Challenging to set up	Easy to set up	Easy to set up

Another widely used model of neurogenesis is two-dimensional (2D) cell culture *in vitro*, including neural stem cell cultures or neuronal cell cultures (Table 1). While cell cultures have the advantage of being easy to maintain and valuable in studying a more homogenous cell population to unravel cellular and molecular mechanisms (Gaspard et al., 2008; Shi et al., 2012), these cellular models have limitations in representing many essential features of the brain. During neurodevelopment, the generation of different types of neurons are temporally controlled and spatially organized. In addition, cell-cell interactions and cytoarchitecture provide external signals that further direct the proliferation, differentiation, migration and circuitry formation during development (Scuderi et al., 2021). These features cannot be modeled in monolayer cultures.

## Brain Organoid Models to Study Neural Stem Cells and Progenitor Cells

**FIGURE 1:**
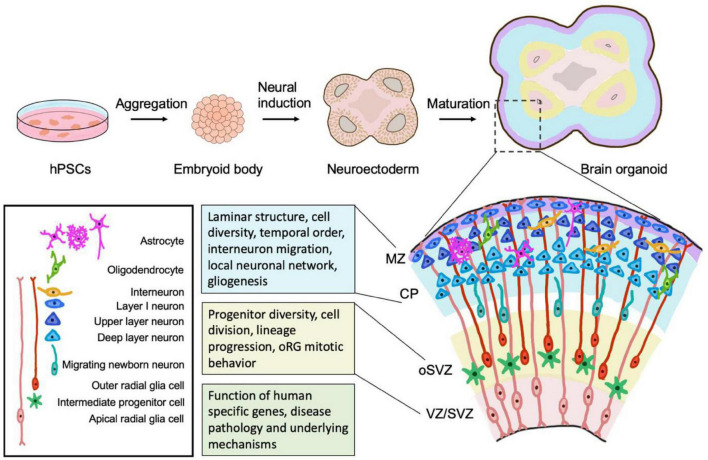
Brain organoids as models to study human neurogenesis. hPSCs are aggregated to form embryoid body and patterned to induce neuroectoderm fate. Neuroectodermal cells organize in the structure of rosette and subsequently develop into brain organoids. Organoids recapitulate the cell diversity and cytoarchitectural organization of the developing human brain. Organoids contain the major progenitor cell pools, including aRGs (apical radial glia cells) in the VZ/SVZ and human specific oRGs (outer radial glia cells) in the oSVZ, with distinct mitotic behavior and lineage progression. Organoids also maintain the structure of the cortical plate in human fetal brain development and thus can be used to investigate the laminar structure formation, generation of principal neuron types (upper layer- and deep layer- neurons), migration, neurogenesis-gliogenesis transition, and the temporal progression and maturation during the neurogenic process. Organoids also provide an opportunity to study the formation of local neuronal networks. Combined with modern genome editing tools and patient-derived iPSCs, organoids can be applied to study the functional contribution of human-specific genes and disease pathology and underlying mechanisms. MZ, marginal zone. CP, cortical plate. VZ, ventricular zone. SVZ, subventricular zone, oSVZ, outer subventricular zone.

The earliest neural stem cells in cortical development are the neuroepithelial cells (NEs) in the ventricular zone (VZ), generated shortly after the formation and closure of neural tube. The initial number and symmetric proliferation of NEs determine the size of the cortex and set the size difference between the mouse and human brain at the very beginning of brain development (Meyer et al., 2000; Haubensak et al., 2004; Lui et al., 2011). At the onset of corticogenesis, NEs transform into apical radial glial cells (aRGs). aRGs reside in the VZ and proliferate to expand the progenitor pool. RGs give rise to intermediate progenitor cells (IPCs) that colonize in the subventricular zone (SVZ) and divide symmetrically to produce pairs of neurons. RGs extend processes to the apical and pial surfaces of the cortex and serve as scaffolds to guide the migration of newly born neurons to the cortical plate. The symmetric and asymmetric division of progenitor cells and migration of neurons give rise to the laminar structure of developing cortex consisting of the VZ, SVZ, intermediate zone, sub-plate, cortical plate, and marginal zone (Meyer et al., 2000; Kosodo et al., 2004; Götz and Huttner, 2005; Lui et al., 2011; Figure 1). Although these processes have been extensively studied in mice, they are not well investigated in the genetic background and cellular context of the human brain. The SVZ notably expanded in primates and humans with two morphologically distinguished regions–inner and outer SVZ (iSVZ and oSVZ). Outer radial glial (oRG) cells are prevalent progenitor cells in oSVZ and predominantly contribute to the expansion and folding or gyrification of the developing human cortex.

Recently developed 3D brain organoid cultures derived from human pluripotent stem cells represent invaluable tools to address questions related to human-specific stem cell types and cytoarchitectures that are difficult to answer in mouse or 2D cell culture models (Table 1). For example, cortical organoids mimic the organization of neural stem cells and progenitor cells in the shape of rosettes (Kadoshima et al., 2013; Lancaster et al., 2013; Lancaster and Knoblich, 2014; Qian et al., 2016). Typical human cortical organoids contain the VZ, iSVZ and oSVZ radial scaffold and diverse RG cell types. At the apical surface of a lumen structure, SOX2^+^ aRGs can be found forming polarized radial structures, whereas TBR2^+^ IPCs are present in the SVZ region (Figure 1). oSVZ is also significantly expanded during organoid development and populated with oRGs characterized by HOPX, FAM107a and tenascin C (TNC) marker expression (Lancaster et al., 2013; Lancaster and Knoblich, 2014; Watanabe et al., 2017; Qian et al., 2020). Much effort has been invested to find out the extent to which organoids recapitulate human neurogenesis, and these studies showed consistently that organoids are capable of generating most brain cell types with similar transcriptomic profiles as their counterparts in the developing human brain (Camp et al., 2015; Quadrato et al., 2017; Amiri et al., 2018; Nascimento et al., 2019). Indeed, cortical organoids have been used to understand mechanisms underlying key aspects of human neurogenesis, such as evolutionary expansion of NE cells, progenitor diversity, cell division modes and lineage progression (Bershteyn et al., 2017; Subramanian et al., 2017; Andrews et al., 2020; Benito-Kwiecinski et al., 2021). Novel insight has been gained into the division pattern of oRGs and the role of the mTOR pathway in regulating oRG cellular morphology, migration, and mitotic behavior (Bershteyn et al., 2017; Andrews et al., 2020). These studies suggest that cortical organoids are a valuable system to study human-specific features and allow further investigation of questions about the molecular mechanisms underlying the transition of aRGs to oRGs and the potency of oRGs in generating diverse neuronal cell populations.

The driver of the evolutionary differences in the cerebral cortex among humans, non-human primates and other mammals has remained elusive. Cortical organoids offer an unprecedented opportunity to study human-specific genes during evolution (Otani et al., 2016; Fiddes et al., 2018; Kanton et al., 2019; Pollen et al., 2019; Benito-Kwiecinski et al., 2021; Liu and Silver, 2021). The human-specific NOTCH2NL gene was found to be highly expressed in RGs. An organoid model showed that NOTCH2NL activates the NOTCH signaling pathway, expands the progenitor population and results in the expansion of the overall organoid size (Fiddes et al., 2018). Organoids derived from human, gorilla, and chimpanzee cells showed that NE cells are the major contributor to human brain expansion. Differences in the cell shape, differentiation capacity, interkinetic nuclear migration and cell cycle length are key factors shaping the developing human brain (Benito-Kwiecinski et al., 2021).

In addition to the most widely used cortical organoids, specific areas of the central nervous system can be modeled by generating organoids using different patterning methods (Jacob et al., 2020). For example, hippocampus organoids show continuous structures consisting of choroid plexus, cortical hem and medial pallium tissues, and recapitulate the cell types and gene expression profiles similar to that of human hippocampus (Kadoshima et al., 2013; Pellegrini et al., 2020). Thalamic and hypothalamic organoids generate typical stem cell types along the developmental trajectory (Qian et al., 2016; Xiang et al., 2019). Midbrain organoids show structurally similar neuromelanin-like granules (Jo et al., 2016). Cerebellar organoids exhibit a layered structure containing cerebellar plate neuroepithelium, deep cerebellar nuclei, Purkinje and granule neurons (Muguruma et al., 2015). Spinal cord organoids generate intermediate and ventral spinal cord-like tissues with somatosensory neurons and spinal motor neurons (Ogura et al., 2018). However, these models sometimes lack the tissue architecture seen *in vivo* and are relatively simplified. Still, these region-specific organoids are emerging as powerful tools for studying neurogenesis in distinct regions of the human nervous system and modeling related disorders.

## Brain Organoid Models to Study Neural Progeny

The cortical plate of the human brain is composed of neurons and glial cells, organized in a laminar structure of six layers. The asymmetric division of aRGs in the VZ/SVZ and symmetric division of oRGs in the oSVZ generate the majority of excitatory neurons that migrate radially to colonize different layers (Götz and Huttner, 2005; Cheung et al., 2010; Lui et al., 2011). The radial migration and graded maturation of neurons in an inside-out sequence in corticogenesis are conserved in mammals. Cajal–Retzius cells form the marginal zone and secrete Reelin for later layer establishment. The early born neurons form the deepest layer VI, characterized by the expression of TBR1. The later born neurons migrate and bypass early neurons to form more superficial layers with projections to different brain regions. Layer V is occupied by CTIP2^+^ neurons that project mostly to spinal cord, whereas layers II-VI are SATB2^+^ intratelencephalic neurons that project within forebrain. Layers II and III pyramidal neurons are also characterized by the expression of BRN1/2 and CUX1/2 (Meyer et al., 2000; Kast and Levitt, 2019; Bhaduri et al., 2021). The extraordinarily diverse inhibitory interneurons in cortex are generated mostly in the medial and caudal ganglionic eminences. They migrate tangentially to form connections with excitatory neurons and integrate to local neural networks (Gorski et al., 2002; Letinic et al., 2002). Although the basic architecture is conserved in human and mouse, the cell number, cell type and gene expression in each compartment are vastly different. The overall neuron population, especially the upper cortical layers, are profoundly expanded in primates and humans. The proportion of GABAergic neurons generated locally or from ganglionic eminences have prominent differences between rodents and primates (Letinic and Rakic, 2001; Anderson et al., 2002).

While human iPSCs can be directed to differentiate into most neuronal types under 2D culture conditions (Gaspard et al., 2008; Shi et al., 2012), the regionally organized columnar and laminar structures are completely lost, and the more complex neural networks are absent. Cortical organoids provide an attractive complement to animal and 2D cell culture models for studying structural organization, cell diversity, as well as the temporal order along the developmental trajectory. The basic laminar structure of cortical layers has been demonstrated in organoid models. At the onset of neuronal differentiation in cortical organoids, early born TBR1^+^ and CTIP2^+^ neurons, as well as Reelin^+^ Cajal–Retzius cells first appear to form a dense neuronal layer. SATB2^+^ and CUX1^+^ upper layer neurons are found at a later stage and localize close to the surface (Lancaster et al., 2013; Lancaster and Knoblich, 2014; Qian et al., 2016, 2020). Single cell sequencing data show that the diversity of neurons generated in organoids is similar to human fetal tissues (Quadrato et al., 2017; Tanaka et al., 2020). The dynamic production, migration and maturation of neuronal populations is also accompanied by neuronal network formation with a surge in electrical activity (Quadrato et al., 2017; Giandomenico et al., 2019; Trujillo et al., 2019). This holds great potential to deepen our understanding of neuronal circuits and network activity in the developing human cortex.

The recent development of assembloid models furthered integration of interneurons to cortical neuronal networks. Several groups have fused human ganglionic eminence organoids and cortical organoids to investigate the migration and integration of interneurons (Bagley et al., 2017; Birey et al., 2017; Xiang et al., 2017). By applying assembloids to model Timothy syndrome, it was found that interneurons carrying the genetic mutation display abnormal migratory saltations, providing insights into the cellular mechanism of this neurodevelopmental disease.

During the later stages of neurogenesis, RGs switch from neurogenic to gliogenic differentiation to generate glial cells, including astrocytes and oligodendrocytes that later populate throughout the cortical layers (Rash et al., 2019). Organoids show promising production of diverse astrocytes (Dezonne et al., 2017; Sloan et al., 2017; Qian et al., 2020). At later stages of organoid cultures, multiple astrocyte subtypes can be found in neural layers, showing morphology and distribution patterns similar to that of the human cerebral cortex (Qian et al., 2020). Transcriptome analysis show that these astrocytes closely resemble their counterparts in fetal tissue and display gradual maturation over time (Sloan et al., 2017). Another important glial cell type, oligodendrocytes, is also present in organoids (Madhavan et al., 2018; Marton et al., 2019). Oligodendrocytes are the myelin-forming cells in the central nervous system that generate myelin to wrap axons to ensure fast signal transmission and provide metabolic support. Multiple studies showed that oligodendrocytes in organoids display similar cellular and molecular features as human oligodendrocytes *in vivo* (Madhavan et al., 2018; Marton et al., 2019; Gordon et al., 2021). These studies demonstrate the applicability of organoids in understanding neural development and neurological disorders at a more complex level.

## Using Organoid Models to Study Developmental Brain Disorders

Cortical organoids recapitulate key features of neurogenesis, and thus represent an ideal model for investigating the etiology of neurodevelopmental disorders. They provide spatial and temporal information about neurogenesis in the context of 3D tissue along the developmental trajectory, bridging the gap between conventional 2D culture and disease pathogenesis. Moreover, organoids generated from patient-derived iPSCs enable modeling of both genetic and idiopathic disorders that are hard to study in animal models. Cumulative studies have used organoids to model genetic brain disorders including autism spectrum disorder (ASD) (Mariani et al., 2015; Wang et al., 2017; Ye et al., 2017; Mellios et al., 2018; Srikanth et al., 2018; Johnstone et al., 2019; Sun et al., 2019; Kang et al., 2021), schizophrenia (Stachowiak et al., 2017; Qian et al., 2020), microcephaly (Lancaster et al., 2013), macrocephaly (Li et al., 2017) and lissencephaly (Bershteyn et al., 2017; Karzbrun et al., 2018). These studies utilize iPSCs generated from patients or human stem cells containing mutations introduced by CRISPR-Cas9 based genome editing. With the advancement of genetic, sequencing and imaging tools, brain organoids facilitated the discovery of etiology and brought novel insight into molecular and cellular mechanisms of these brain disorders. For example, some ASD organoids exhibit accelerated cell cycle and overproduction of GABAergic inhibitory neurons, indicating disrupted excitatory-inhibitory neuronal networks in these patients. Further investigation revealed the transcription factor FOXG1 is responsible for the overproduction of GABAergic neurons and its expression is correlated with the severity of the disease (Mariani et al., 2015). In an Angelman syndrome organoid model, the mechanism of synaptic dysfunction caused by ubiquitin protein ligase E3A (UBE3A) was illuminated. UBE3A leads to degradation of calcium- and voltage-dependent big potassium channels and suppresses neuronal hyperexcitability. These disease models also allow researchers to test drug treatments based on phenotypes and to facilitate translational studies (Kang et al., 2021).

Organoids are also a valuable model for studying brain infectious diseases (Cugola et al., 2016; Nowakowski et al., 2016; Qian et al., 2016; Watanabe et al., 2017; Jacob et al., 2020). For example, utilizing organoid models, researchers found that ZIKV causes progenitor cell death and reduced proliferation, resulting in a microcephaly-like phenotype (Dang et al., 2016; Qian et al., 2016). In addition to uncovering the infection mechanism, organoids also facilitated drug design and screening to mitigate the damage of ZIKV on the developing brain (Dang et al., 2016; Watanabe et al., 2017). Together, this recent progress in applying organoids to multi-disciplinary research suggest that brain organoids provide a powerful platform for uncovering the etiology of neural developmental disorders, investigating disease mechanisms and testing drug efficacy for better treatments.

## Limitations

While valuable insights into neurogenesis have been gained from brain organoid studies, there are limitations of this *in vitro* model in fully recapitulating human brain development. Although organoids have shown advantages in regard to diverse progenitor and neuron types and the fidelity of organoid models has been heavily investigated by comparing the cell types and gene expression at the single cell level in the fetal brain, not all cellular subtypes are present and certain cells show altered gene expression at the molecular level (Amiri et al., 2018; Pollen et al., 2019; Jacob et al., 2021). Thus, further improvement of organoid protocols and culture conditions is needed to better recapitulate neurogenesis in the human brain.

Microglia, a resident immune cell type in the brain, is absent in cortical organoids due to their non-neural lineage. In rodent models, microglia have been shown to play essential roles in neurogenesis of the developing cortex. They adapt their functions in diverse states to regulate programmed cell death and synapse elimination, and have a profound impact on maintaining homeostasis in the brain (Cunningham et al., 2013; Butovsky and Weiner, 2018; Li and Barres, 2018). Thus, it would be beneficial to integrate microglia into brain organoids to better mimic the physiological environment and to study immune response in infectious neurological diseases. Indeed, several groups have attempted to differentiate microglia from stem cells or iPSCs and incorporate them into organoids (Ormel et al., 2018; Song et al., 2019; Xu et al., 2021). Another approach is transplantation of organoids into mouse brains. Progressive microglia integration from the host can be found in human organoid grafts in mouse cortex (Mansour et al., 2018). These methods provide great potential to broaden our understanding of the complexity of neurogenesis under normal and disease conditions.

Following the expansion of organoids during culture, a hypoxic necrotic core inevitably develops due to a lack of vascularization and inefficient oxygen and nutrient exchange. Single cell sequencing data also revealed upregulated glycolytic and ER stress genes in organoids (Amiri et al., 2018; Bhaduri et al., 2020). These cellular stressors are postulated to impair cellular diversity and neuronal differentiation. In addition, endothelial cells from vasculature are crucial for establishing a niche that stimulates the self-renewal of neural stem cells and IPCs (Qin et al., 2004; Wang et al., 2019). Vascularization can be established after engrafting organoids into the mouse brain, as mouse endothelial cells could invade human organoids and establish vasculature for nutrient supply (Daviaud et al., 2018; Mansour et al., 2018). Direct generation of vascularized organoids *in vitro* has also been attempted, while some features of the human fetal telencephalon can be found, delivery of oxygen and nutrients through the vasculature has not been achieved *in vitro* (Shi et al., 2020). Successful integration of vascular structure with active flow for nutrient exchange in organoids will not only support better survival, but also facilitate the homeostasis of many cell types, and bring new perspectives on human neurogenesis.

Another major caveat of the organoid model is the variation of morphology and inconsistency. This is caused by differences in the genetic background of stem cells and iPSCs, as well as methods of patterning and culturing. Although brain organoids display some hallmarks of structure and cell type diversity, their size and morphology vary dramatically, generating different qualities and quantities within and between batches. In addition, protocols for organoids differentiation are different across research groups, raising the question of whether the developmental trajectories and cell identities are equivalent using different methodologies. Many groups have addressed the heterogeneity of organoids by transcriptome comparison (Pollen et al., 2019; Velasco et al., 2019; Yoon et al., 2019). However, it is still a challenge to establish a universal organoid differentiation protocol with reproducibility and robustness.

In summary, brain organoids are valuable tools to study brain development and have greatly expanded our toolbox and knowledge of human neurogenesis. With further improvement in organoid technology and incorporation of bioengineering and molecular tools, such as engineered scaffolds, optogenetics and chemical genetics, and synaptic tracing, more advanced experiments can be performed using organoid models to generate novel insights into neurogenesis in the human brain.

## Data Availability Statement

The original contributions presented in the study are included in the article/supplementary material, further inquiries can be directed to the corresponding author/s.

## Author Contributions

QY contributed the figure and the table. All authors contributed to the article and approved the submitted version.

## Conflict of Interest

The authors declare that the research was conducted in the absence of any commercial or financial relationships that could be construed as a potential conflict of interest.

## Publisher’s Note

All claims expressed in this article are solely those of the authors and do not necessarily represent those of their affiliated organizations, or those of the publisher, the editors and the reviewers. Any product that may be evaluated in this article, or claim that may be made by its manufacturer, is not guaranteed or endorsed by the publisher.
